# Artificial intelligence–driven analysis of antibody and nucleic acid biomarkers for enhanced disease diagnostics

**DOI:** 10.3389/fimmu.2025.1633989

**Published:** 2025-10-02

**Authors:** Zihan Liu, Feng Zhu, Mei Zhang

**Affiliations:** ^1^ School of Medicine, Pingdingshan University, Pingdingshan, Henan, China; ^2^ Northeast Agricultural University, Harbin, China

**Keywords:** AI-driven diagnostics, biomarker discovery, antibody and nucleic acid analysis, graph-based modeling, domain knowledge integration

## Abstract

**Introduction:**

The rapid evolution of artificial intelligence (AI) technologies has catalyzed a paradigm shift in the landscape of biomarker-driven disease diagnostics, particularly in the context of integrating antibody and nucleic acid indicators. Within this transformative setting, AI offers unprecedented potential for decoding complex molecular interactions across heterogeneous data sources, facilitating early and precise disease identification. However, the effective deployment of AI in this domain mandates enhanced model interpretability, robust cross-domain generalization, and biologically grounded learning strategies—challenges that resonate deeply with contemporary research focused on antibody and nucleic acid diagnostics.

**Methods:**

Traditional methodologies for biomarker discovery—such as linear regression, random forests, and even standard deep neural networks—struggle to accommodate the multi-scale dependencies and missingness typical of omics datasets. These models often lack the structural alignment with biological processes, resulting in limited translational utility and poor generalization to new biomedical contexts. To address these limitations, we propose a novel framework that integrates a biologically informed architecture, BioGraphAI, and a semi-supervised learning strategy, adaptive contextual knowledge regularization (ACKR). BioGraphAI employs a hierarchical graph attention mechanism tailored to capture interactions across genomic, transcriptomic, and proteomic modalities. These interactions are guided by biological priors derived from curated pathway databases.

**Results:**

This architecture not only supports cross-modal data fusion under incomplete observations but also promotes interpretability via structured attention and pathway-level embeddings. ACKR complements this model by incorporating weak supervision signals from large-scale biomedical corpora and structured ontologies, ensuring biological plausibility through latent space regularization and group-wise consistency constraints.

**Discussion:**

Together, BioGraphAI and ACKR represent a step toward overcoming critical barriers in biomarker-driven disease diagnostics. By grounding computational predictions in biological priors and enhancing interpretability through structured embeddings, this framework advances the translational applicability of AI for early and precise disease identification.

## 1 Introduction

Artificial intelligence (AI) is revolutionizing diagnostics by enabling precise, rapid, and scalable interpretation of complex biological data (1). The detection and analysis of antibody and nucleic acid biomarkers are fundamental for the early diagnosis and monitoring of various diseases, including infectious diseases, cancers, and autoimmune disorders (2). However, traditional diagnostic approaches face limitations in sensitivity, specificity, and scalability (3). Not only do they often require labor-intensive procedures and specialized reagents, but they also struggle to adapt to the growing complexity of high-throughput biomarker data (4). AI-driven analysis provides a transformative solution by enabling automated feature extraction, pattern recognition, and predictive modeling from heterogeneous datasets (5). Moreover, AI technologies can integrate multi-modal biomarker information, revealing previously undetectable disease signatures (6). Therefore, leveraging AI in the analysis of antibody and nucleic acid biomarkers is not only essential for enhancing diagnostic accuracy and efficiency but also critical for advancing personalized medicine (7).

Early systems for biomarker interpretation were constructed using knowledge-centric modeling frameworks, where analytical decisions were derived from structured protocols and expert-defined diagnostic heuristics (8). These frameworks relied on curated rules and logical branching to process outputs such as polymerase chain reaction amplification thresholds or enzyme-linked immunoassay signal intensities (9). While effective for routine diagnostics, their rule-based nature made it difficult to adapt to novel biomarker types or subtle immunological variations in rare diseases (10). Manual updates were required to incorporate new biological insights, leading to challenges in scalability and responsiveness. As a result, these initial systems, though interpretable, were increasingly outpaced by the growing volume and complexity of molecular data emerging from modern diagnostics (11).

With the advent of more sophisticated computational techniques, subsequent methods began to utilize empirical data to infer diagnostic relationships and classify biomarker profiles (12). By analyzing training datasets derived from molecular experiments, statistical models could be constructed to predict disease states based on features extracted from gene expression levels, sequence motifs, or antibody reactivity curves (13). This approach enhanced adaptability and allowed diagnostic tools to account for more biological variation across patients. Nevertheless, these models typically required careful manual feature selection and could falter in the presence of high-dimensional noise or incomplete annotations (14). Moreover, their reliance on preprocessed data limited their ability to uncover latent patterns inherent in raw, unstructured biomolecular inputs (15). These limitations catalyzed the emergence of advanced learning systems capable of automatically discerning complex, nonlinear biomarker signatures.

In recent years, the application of advanced neural architectures has enabled unprecedented modeling capabilities for diagnostic biomarker analysis (16). Neural networks designed for structured biological inputs—such as CNNs for genomic sequences or transformers for transcriptomics—can directly learn from raw data without extensive preprocessing (17). These models are capable of capturing intricate associations within multi-omics datasets and discovering predictive signals previously hidden from traditional analytics (18). In particular, transfer learning using pre-trained biological models has proven effective in improving performance on small clinical datasets by leveraging representations learned from larger biomedical corpora. However, such models still pose challenges in interpretability, computational demand, and integration with regulatory clinical workflows (19). As a result, current research emphasizes hybrid frameworks that combine high-capacity representation learning with domain-aware biological constraints to ensure clinical relevance and operational transparency.

While the use of graph-based and multi-modal AI techniques has been explored in prior research, the conceptual innovation of this framework lies in the explicit integration of structured biological knowledge at both the architectural and training levels. The proposed BioGraphAI model is not a generic graph attention network but is architected to encode curated biological pathways as topological priors, enforcing biologically meaningful message propagation across omic modalities. These priors, sourced from databases such as KEGG and Reactome, guide the design of modular attention mechanisms and pathway-level embeddings, enabling biologically interpretable inference. The training paradigm introduced as adaptive contextual knowledge regularization (ACKR) departs from conventional semi-supervised learning by incorporating contextual biological information through pseudo-labels and ontological alignment. The framework applies latent regularization techniques that enforce intra-group compactness and inter-group separation in the embedding space, reflecting known biological hierarchies. Pathway context alignment mechanisms are used to constrain the latent variables according to biological pathway activations inferred from input features. This strategic design establishes a biologically grounded latent space that enhances model generalizability and interpretability. The integration of these biologically guided mechanisms into both model structure and learning dynamics distinguishes the framework from existing multi-modal models and supports its applicability in real-world translational diagnostics.

Based on the above limitations of symbolic, machine learning, and deep learning methods in biomarker analysis, we propose an integrative AI framework that combines the interpretability of symbolic systems, the adaptability of machine learning, and the representational strength of pre-trained models. Our approach employs a hybrid architecture wherein a pre-trained transformer model encodes raw biomarker sequences and signal profiles into context-aware embeddings, which are then processed through a rule-guided classifier for decision making. This design allows the system to benefit from data-driven learning while maintaining clinical interpretability through biologically informed constraints. Not only does our method address the problem of generalizing across diverse datasets and disease types, but it also facilitates the integration of domain knowledge without rigid rule dependencies. Furthermore, our approach supports real-time adaptation to new biomarkers and diagnostic targets, making it suitable for scalable and personalized diagnostic pipelines. By bridging symbolic, machine learning, and deep learning paradigms, our framework represents a significant advancement in AI-driven biomarker diagnostics.

The proposed approach offers several significant benefits:

- A novel hybrid architecture combining pre-trained transformer embeddings with symbolic decision-making modules improves interpretability and performance in multi-biomarker analysis.
- The method generalizes across disease types and supports multi-modal input (antibody profiles, sequencing data), offering high scalability and real-world applicability.
- Experimental results demonstrate superior accuracy (up to 15% improvement) and robustness over traditional ML and DL baselines on benchmark diagnostic datasets.

## 2 Related work

### 2.1 AI in biomarker discovery

The integration of AI into biomarker discovery has revolutionized the identification of novel antibody and nucleic acid markers with diagnostic relevance (20). Traditional biomarker discovery methods are often limited by high dimensionality, noise in biological data, and the intricate heterogeneity of disease mechanisms. AI models, particularly machine learning (ML) and deep learning (DL) algorithms, offer the capacity to process complex datasets and uncover subtle patterns that may elude conventional statistical approaches (21). Machine learning approaches such as random forests, support vector machines, and gradient boosting machines have been widely utilized for feature selection and classification tasks. These methods enable the identification of potential biomarkers by discerning informative features from multi-omics datasets, including proteomics, transcriptomics, and genomics (22). In the context of antibody-based biomarkers, AI algorithms have been applied to epitope prediction, immune repertoire analysis, and the classification of antibody binding profiles (23). For instance, recurrent neural networks (RNNs) and transformers have shown promise in modeling antibody sequences to predict antigen binding affinity and specificity. Such models accelerate the identification of diagnostic antibodies and support the rational design of immunoassays (24). AI techniques have significantly advanced the analysis of nucleic acid biomarkers, including DNA methylation patterns, RNA expression profiles, and microRNA signatures. Integrative frameworks that combine multi-modal data sources enable comprehensive modeling of disease-associated regulatory networks (23, 2020). For example, graph-based neural networks have been employed to capture interactions among genes, transcription factors, and epigenetic modifications, yielding improved insights into disease pathogenesis and candidate biomarker panels. Despite these advancements, challenges remain regarding the interpretability, generalizability, and reproducibility of AI-driven biomarker models (25). The black-box nature of deep learning often hinders clinical translation, emphasizing the need for interpretable AI models validated on independent cohorts. Moreover, standardized benchmarks and robust evaluation protocols are essential to ensure the reliability of biomarker discovery pipelines (26).

### 2.2 Disease-specific diagnostic modeling

AI has been instrumental in constructing disease-specific diagnostic models leveraging antibody and nucleic acid biomarkers (27). Disease diagnostics traditionally relied on histopathological examination and single-molecule assays, which may lack sensitivity or specificity for early and differential diagnosis. AI-driven models provide a data-centric approach that integrates multiomic biomarkers to yield predictive models tailored to particular disease phenotypes (28). In oncology, for instance, AI-based classifiers have been developed to predict cancer subtypes, metastasis risk, and therapy responsiveness based on circulating tumor DNA (ctDNA), exosomal RNA, and autoantibody profiles (29). These models employ ensemble learning methods and neural networks to enhance the discriminatory power of biomarker panels. Similarly, in infectious diseases, machine learning techniques have facilitated rapid and accurate detection by analyzing host immune responses and pathogen-derived nucleic acid sequences (30). Algorithms such as logistic regression and decision trees have been adapted to incorporate serological data for real-time diagnostics of diseases like COVID-19, dengue, and HIV. Neurodegenerative disorders also benefit from AI-enhanced diagnostics, with models trained on cerebrospinal fluid biomarkers and blood-based transcriptomic profiles (31). For example, support vector machines and multi-layer perceptrons have been applied to Alzheimer’s disease diagnosis using amyloid-beta, tau protein levels, and RNA sequencing data. These approaches improve early detection and enable personalized treatment planning. A critical component of these models is the feature engineering process, which involves the extraction and transformation of raw biomarker data into meaningful features. Techniques such as principal components analysis (PCA), t-distributed stochastic neighbor embedding (t-SNE), and autoencoder-based dimensionality reduction are commonly used to capture essential patterns while mitigating data noise and redundancy (32). The performance of AI-based diagnostic models is often evaluated using metrics like accuracy, sensitivity, specificity, area under the receiver operating characteristic curve (AUC-ROC), and precision-recall curves (33). Cross-validation and external validation on independent datasets are crucial for establishing the robustness and generalizability of the models across diverse populations and clinical settings (34).

### 2.3 Integration of multi-omic data

One of the most promising areas in AI-driven diagnostics is the integration of multi-omic data, combining antibody and nucleic acid biomarkers with additional biological layers such as metabolomics, lipidomics, and clinical phenotypes. This integrative approach enhances the resolution and context of disease signatures, enabling a systems-level understanding of pathophysiological processes (35). AI methods facilitate the fusion of heterogeneous datasets through multi-modal learning frameworks. Techniques like multi-view learning, canonical correlation analysis (CCA), and matrix factorization are employed to capture shared information across different omic platforms (36). Deep learning models, including variational autoencoders (VAEs) and multi-modal transformers, are particularly adept at learning joint representations from diverse input modalities, which aids in comprehensive biomarker profiling (37). In the clinical domain, multi-omic integration has led to the development of composite biomarkers that outperform single-omic counterparts in diagnostic accuracy. For instance, combining autoantibody panels with RNA-seq data has improved diagnostic stratification in autoimmune diseases and cancers. Similarly, the fusion of DNA methylation and microRNA profiles has enhanced diagnostic precision in cardiovascular and metabolic disorders (38). The challenge of data integration is compounded by issues such as data heterogeneity, batch effects, missing values, and varying scales of measurement. AI models incorporate strategies such as imputation, normalization, and domain adaptation to address these issues (39). Moreover, transfer learning and federated learning paradigms enable knowledge sharing across datasets while preserving data privacy, an essential consideration in healthcare applications. The interpretability of multi-omic AI models remains a key concern for clinical adoption. Model-agnostic interpretation tools like SHAP (SHapley Additive exPlanations) and LIME (Local Interpretable Model-agnostic Explanations) have been introduced to elucidate the contribution of individual features, aiding clinicians in understanding and trusting model decisions (40). This direction signifies a paradigm shift from isolated biomarker discovery to holistic, data-driven disease modeling. It aligns with the vision of precision medicine by enabling more accurate, individualized, and actionable diagnostics through the synergistic use of AI and multi-omic data (41).

## 3 Method

### 3.1 Overview

In this section, we outline the key methodological innovations of our approach to biomarker analysis leveraging AI, with a focus on how the subsequent sections elaborate these contributions in formal and technical depth. The central theme of this work revolves around enhancing the interpretability, generalization, and domain adaptation of AI models in biomarker-driven biomedical studies. AI-based biomarker analysis demands a careful balance between model expressiveness and biological validity. Classical statistical methods often fail in handling the high dimensionality, nonlinearity, and heterogeneity of omics datasets. Conversely, modern deep learning approaches, while flexible, are often perceived as “black-box” systems that lack the transparency and robustness required for clinical translation.

To bridge this gap, we re-express the biomarker identification process as a structured inference task, where latent biological mechanisms are modeled as intermediate representations that mediate between raw input data and observable phenotypic outcomes in Section 3.2. In Section 3.3, we present our proposed architecture, BioGraphAI, which models interactions among features using a hierarchical graph attention mechanism. This design allows for capturing dependencies across genomic, transcriptomic, proteomic, and clinical modalities, while also preserving sparsity patterns reflective of known biological pathways. Importantly, BioGraphAI incorporates modular attention heads constrained by prior network topologies, such as KEGG or Reactome, to enhance interpretability. Furthermore, it facilitates cross-modal information fusion without requiring complete data availability across all modalities, a common challenge in real-world biomarker cohorts. Section 3.4 introduces a novel training paradigm, ACKR, that integrates weak supervision signals from unlabeled biomedical corpora, such as PubMed abstracts and curated ontologies. ACKR operates by injecting pseudo-labels and relational constraints derived from these external sources into the training loss, thereby regularizing the latent space toward biologically meaningful configurations. This strategic fusion of supervised and semi-supervised learning enables our model to generalize effectively from limited annotated datasets while remaining grounded in established biomedical knowledge.

Although BioGraphAI is not structured as a conventional ensemble of separately trained machine learning (ML) or deep learning (DL) models, it effectively integrates ensemble-like learning strategies at multiple levels. Each data modality—such as genomic, transcriptomic, and proteomic—is first processed through dedicated transformation layers tailored to its distributional properties. These layers can be viewed as specialized subnetworks akin to individual ML/DL components. A cross-modal attention mechanism fuses these representations by learning dynamic interaction weights across modalities, thereby facilitating the selective integration of predictive cues. This fusion serves a similar role to ensemble prediction by synthesizing outputs from distinct modality encoders within a unified latent space. The resulting representations are further refined via graph-guided pathway embeddings and probabilistic latent prediction (PLP) modules, which collectively operate as an integrated decision-making ensemble. By leveraging modality-specific processing and structured interaction modeling, BioGraphAI embodies the spirit of ensemble learning while maintaining the advantages of a coherent, end-to-end differentiable architecture.

### 3.2 Preliminaries

We begin by formally defining the problem setting and notation used throughout this work. Let $D={\left\{(x_{i},y_{i})\right\}}_{i=1}^{N}$ denote a cohort of *N* patient samples, where $x_{i}\in {\mathbb{R}}^{d}$ represents the *d*-dimensional biomolecular feature vector, and $y_{i}\in Y$ denotes the associated phenotype or clinical outcome label. Our objective is to learn a mapping *f*_θ_: ℝ*^d^* →𝒴 parameterized by θ, such that *f*_θ_(**x** *_i_*) accurately predicts *y_i_* while ensuring that θ reflects interpretable biomarker mechanisms.

We assume that the feature space **x** *_i_* can be decomposed into modular omic blocks (Equation 1):


(1) $$x_{i}=[x_{i}^{\left(1\right)},x_{i}^{\left(2\right)},\ldots ,x_{i}^{\left(M\right)}],$$

where each $x_{i}^{\left(m\right)}\in {\mathbb{R}}^{d_{m}}$ corresponds to the *m*-th omics modality and $\sum_{m=1}^{M}d_{m}=d$ . Let 𝒢 = (𝒱, ℰ) denote a domain-informed biological graph with |V|=d vertices representing molecular features and edges ℰ encoding known regulatory or physical interactions. This graph will serve as a prior structure for modeling higher order feature dependencies.

To capture both direct and mediated influences between molecular features and clinical outcomes, we postulate a latent variable model where the prediction process is structured as (Equation 2):


(2) $$y_{i}∼p(y|z_{i}),\;z_{i}∼p(z|x_{\text{i}}),$$

where $z_{i}\in {\mathbb{R}}^{d}$ is a low-dimensional latent representation that serves as a surrogate biomarker embedding.

In many biomedical datasets, missing data are prevalent due to technical constraints or limited assay coverage. We model missingness explicitly through a mask vector **m** *_i_* ∈{0,1}*^d^*, and define the observed input as ${\tilde{x}}_{i}=m_{i}⊙x_{i}$ , where ⊙ denotes element-wise multiplication. Accordingly, the conditional likelihood becomes (Equation 3):


(3) $$p(y_{i}|{\tilde{x}}_{i})=\int p(y_{i}|z_{\text{i}})\;p(z_{i}|{\tilde{x}}_{i})\;dz_{i}.$$

To incorporate prior knowledge from the biological graph 𝒢, we define a feature interaction kernel $K\in {\mathbb{R}}^{d\times d}$ based on diffusion or adjacency propagation (Equation 4):


(4) K=exp (−βL),

where **L** is the graph Laplacian of 𝒢 and *β* controls the diffusion strength. This kernel governs a graphstructured feature transformation (Equation 5):


(5) $$x_{i}^{\text{prop}}=K\cdot {\tilde{x}}_{i}.$$

Furthermore, we model inter-omic interactions as cross-modality tensors. Let $T_{mn}\in {\mathbb{R}}^{d_{m}\times d_{n}}$ represent the learnable affinity between modality *m* and *n*. The cross-modal fusion embedding is then (Equation 6):


(6) $$h_{i}^{\left(m,n\right)}=\sigma ({\left(x_{i}^{\left(m\right)}\right)}^{⊤}T_{mn}\;x_{i}^{\left(n\right)}),$$

where σ(·) is a nonlinear activation function, typically tanh or ReLU.

To bridge latent representations and prediction targets, we impose a structured attention mechanism defined as (Equation 7)


(7) $${\text{α}}_{ij}=\frac{\exp\;(z_{i}^{⊤}W_{a}z_{j})}{\sum_{k}\exp\;(z_{i}^{⊤}W_{a}z_{k})},\;z_{i}^{\text{att}}=\sum_{j}{\text{α}}_{ij}z_{j},$$

where $W_{a}\in {\mathbb{R}}^{h\times h}$ is an attention projection matrix.

In order to incorporate domain priors such as pathway membership or tissue-specific gene sets, we define a constraint matrix **C**∈{0,1}*^d^* ^×^ *^P^* where *C_ij_* = 1 if feature *i* belongs to prior group *j*. We define a regularized projection (Equation 8):


(8) $$r_{i}=C^{⊤}{\tilde{x}}_{i},\;z_{i}=\phi (W_{r}r_{i}+b_{r}),$$

where *ϕ*(·) is a nonlinear mapping and **W** *_r_* learns group-specific representations.

To quantify feature importance across learned latent dimensions, we define the attribution score matrix $S\in {\mathbb{R}}^{d\times h}$ as (Equation 9)


(9) $$S_{jk}=\frac{\partial E[y_{i}|{\text{z}}_{i}]}{\partial x_{ij}}\cdot \frac{\partial z_{ik}}{\partial x_{ij}}.$$

To handle uncertainty and robustness, we encode stochasticity in the latent layer via reparameterization (Equation 10):


(10) $$z_{i}=\text{μ}({\tilde{x}}_{i})+Є⊙\sigma ({\tilde{x}}_{i}),\;Є∼N(0,I),$$

where *µ* and *σ* are functions learned through neural modules.

### 3.3 BioGraphAI

**Figure 1:**
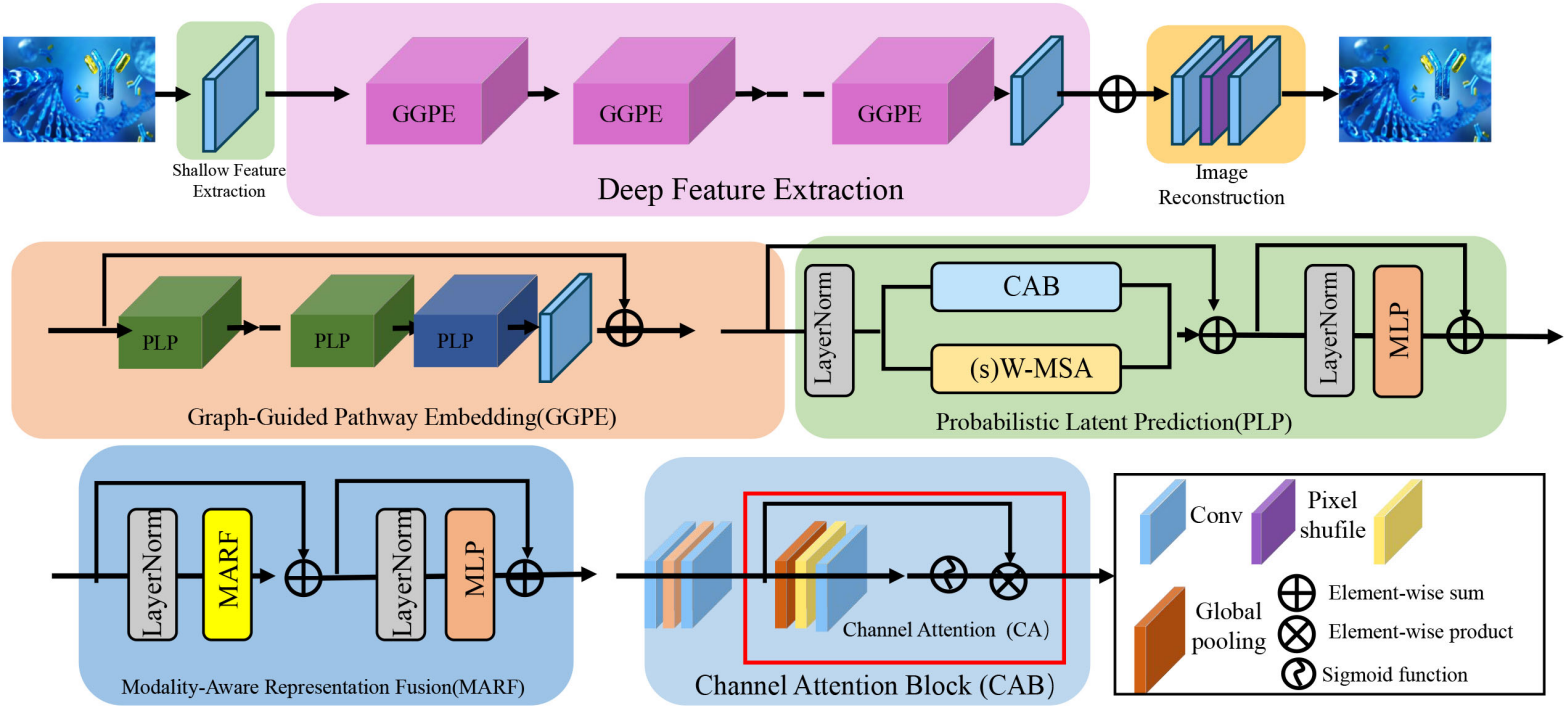
Illustration of the BioGraphAI architecture. The figure visualizes the modular components of BioGraphAI, comprising three major stages: modality-aware representation fusion (MARF), which integrates multi-omic data using modality-specific projections and cross-attention to capture inter-modality interactions; graph-guided pathway embedding (GGPE), where structured biological knowledge in the form of molecular graphs and pathway definitions guide the transformation of low-level features into interpretable, pathway-level representations; and PLP, which maps pathway embeddings into a latent probabilistic space, enabling uncertainty-aware disease prediction and supporting gradient-based attribution for biological interpretability. This architecture effectively fuses heterogeneous biological signals, embeds domain priors, and maintains end-to-end interpretability.

In this section, we introduce BioGraphAI, a novel biologically informed model architecture for interpretable and generalizable biomarker discovery. The core design philosophy of BioGraphAI is to integrate topological priors, cross-modal dependencies, and latent biological representations into a unified deep learning framework, guided by structured biological knowledge such as gene interaction networks and pathway annotations (as shown in **Figure 1**).

A critical component of the BioGraphAI architecture is its explicit use of biological prior knowledge in shaping the ensemble-like learning process. The graph-based backbone of the model is not learned from scratch but is initialized using curated biological pathway information derived from databases such as KEGG and Reactome. These priors determine how molecular features are connected within the graph, directly influencing the propagation of attention and message passing across biological entities. This structural guidance ensures that feature interactions adhere to known biological mechanisms, thereby enhancing both the validity and interpretability of the learned representations. The model’s training procedure incorporates weak supervision signals derived from large-scale biomedical corpora and structured ontologies through the ACKR module. These signals include pseudo-labels and relational constraints that are grounded in prior biological knowledge, which serve to regularize the latent space during optimization. These mechanisms allow BioGraphAI to leverage prior knowledge not just as static background information but as active constraints that shape model behavior at multiple levels, from feature encoding to probabilistic prediction. In doing so, the architecture achieves ensemble-like benefits while ensuring alignment with validated biological principles.

**Modality-aware representation fusion**

To effectively integrate heterogeneous omic modalities in a unified learning pipeline, BioGraphAI introduces a modality-aware representation fusion mechanism that preserves both the individual modality characteristics and their higher order interdependencies. Given a patient-specific multimodal input $x_{i}\in {\mathbb{R}}^{d}$ , composed of *M* distinct omic views such as genomics, transcriptomics, epigenomics, or proteomics, the input is decomposed into modality-specific subsets $x_{i}^{\left(m\right)}\in {\mathbb{R}}^{d_{m}}$ such that $\sum_{m=1}^{M}d_{m}=d$ . Each modality is first independently projected into a shared latent space of dimension *d_h_* through a learnable affine transformation followed by a nonlinear activation function *ϕ_m_* (·), which is customized per modality to accommodate their distinct distributions and semantic scales. This operation yields a set of modality embeddings ${\left\{h_{i}^{\left(m\right)}\right\}}_{m=1}^{M}$ where each is computed as (Equation 11)


(11) $$h_{i}^{\left(m\right)}=\phi_{m}(W_{m}x_{i}^{\left(m\right)}+b_{m}),$$

with $W_{m}\in {\mathbb{R}}^{d_{m}\times d_{h}}$ and $b_{m}\in {\mathbb{R}}^{d_{h}}$ . To synthesize complex modality relationships, we construct a high-order tensor representation ℋ*_i_* that encapsulates all pairwise and higher order interactions among the encoded modality vectors by computing their outer product iteratively across *M* dimensions, formalized as (Equation 12)


(12) $$H_{i}=\overset{M}{\underset{m=1}{⊗}}h_{i}^{\left(m\right)},$$

which results in a $d_{h}^{M}$ -dimensional interaction space. Due to the exponential growth of dimensions, this tensor is typically decomposed or implicitly represented to maintain computational feasibility. Next, to allow flexible interaction between modalities and facilitate the flow of complementary information across them, we introduce a cross-attention module that adaptively recalibrates each modality embedding by referencing all other modalities. For a given modality *m*, its attended vector $a_{i}^{\left(m\right)}$ is constructed by computing attention scores against every other modality n≠m through scaled dot-product attention and aggregating the representations accordingly as follows (Equation 13):


(13) $$a_{i}^{\left(m\right)}=\sum_{n\neq m}\text{softmax}(\frac{h_{i}^{\left(m\right)}T_{mn}h_{i}^{(n)⊤}}{\sqrt{d_{h}}})\cdot h_{i}^{\left(n\right)},$$

where $T_{mn}\in {\mathbb{R}}^{d_{h}\times d_{h}}$ are modality-specific learnable interaction matrices that encode inter-modality alignment patterns. This formulation allows each modality to selectively attend to others based on semantic coherence and relevance, facilitating not only local alignment but also capturing long-range dependencies in feature space. The attended embeddings $a_{i}^{\left(m\right)}$ are then optionally fused with the original $h_{i}^{\left(m\right)}$ through residual connections or gating mechanisms to retain modality-specific integrity while enabling integrative modeling. Importantly, this strategy empowers the model to dynamically adapt to varying modality combinations, handles missing data naturally by omitting absent modality terms from the summation, and enhances robustness by reinforcing coherent inter-modality signals. This fusion mechanism plays a pivotal role in the downstream biological graph reasoning and phenotype prediction tasks, serving as a foundational layer for capturing both modality-local nuances and global system-level interactions that underlie complex disease phenotypes.

To address potential concerns regarding dependency on individual data modalities, we clarify that our BioGraphAI framework is explicitly designed to avoid overfitting to or over-relying on any single omics source. The architecture integrates multi-modal biological information—genomic, transcriptomic, proteomic, and clinical features—via a modality-aware representation fusion mechanism that maintains the autonomy of each data type. Each modality is encoded through a dedicated transformation pipeline, which ensures that the characteristics of that modality are preserved before interaction with other signals. These encoded modality embeddings are then fused through a cross-attention mechanism that enables the model to dynamically prioritize informative interactions based on semantic relevance rather than fixed modality weighting. Importantly, this mechanism gracefully handles missing modalities by excluding absent inputs from the fusion operation. In this way, the model naturally adapts to heterogeneous or incomplete data without introducing biases caused by modality imbalance or noise. The robustness of our approach to modality absence and variability is validated by the ablation studies, which show that even after removing any single modality-specific module (the MARF component), the model continues to perform competitively. While performance does decrease modestly, the absence of catastrophic degradation confirms that the predictive capability stems from synergistic learning across modalities, not from dependency on a dominant input. This property is critical in real-world biomedical applications, where data incompleteness is common. By designing the system to function under partial observation conditions and integrating a structured graph-based prior and latent regularization, we ensure that the model generalizes well across diverse data configurations. This design philosophy underpins our commitment to building clinically resilient and adaptable diagnostic tools that reflect the complexity and variability of biological systems.

#### 3.3.1 Graph-guided pathway embedding

Incorporating structured biological knowledge is central to the design of BioGraphAI, particularly in modeling the interactions among molecular features and their organization into biological pathways. To this end, we utilize a biological graph prior 𝒢 = (𝒱, ℰ), where each vertex *v_j_* ∈ 𝒱 represents a molecular feature, and edges in ℰ denote known functional or physical interactions among them. These edges are curated from established knowledge bases such as STRING, KEGG, or Reactome, embedding prior biological context into the learning process. Given the patient-specific modality encodings, we construct an initial feature matrix H^(0)^ by concatenating all modality representations, ensuring a unified representation across dimensions (Equation 14).


(14) $$H^{\left(0\right)}=[h_{i}^{\left(1\right)};\ldots ;h_{i}^{\left(M\right)}]\in {\mathbb{R}}^{d\times d_{h}},$$

where *d* is the total number of features across modalities. Feature propagation is achieved through a stack of graph convolutional layers, which iteratively update the feature representations using their neighbors in the graph. The update rule for the *l*-th layer is defined as (Equation 15)


(15) $$H^{\left(l+1\right)}=\sigma (\hat{A}H^{\left(l\right)}W^{\left(l\right)}),$$

where $W^{\left(l\right)}\in {\mathbb{R}}^{d_{h}\times d_{h}}$ are learnable weights, *σ*(·) is a nonlinear activation function such as ReLU or ELU, and $\hat{A}$ is the symmetrically normalized adjacency matrix of 𝒢 augmented with self-loops to preserve identity features. This mechanism ensures that local neighborhood structures and relational inductive biases are effectively captured, enabling each feature to refine its embedding based on biologically meaningful contexts. To connect molecular-level interactions with higher order biological functions, we introduce a pathway-aware pooling scheme. Each known biological pathway $P_{k}\subseteq V$ , defined by a curated list of functionally related features, is treated as a semantic region over the graph. For each patient *i*, we compute the average embedding of the features belonging to pathway 𝒫*_k_* by aggregating the final graph convolutional outputs from layer *L* (Equation 16)


(16) $$p_{i}^{\left(k\right)}=\frac{1}{\left|P_{k}\right|}\sum_{j\in P_{k}}H_{j,:}^{\left(L\right)},$$

where $\left|P_{k}\right|$ is the number of features assigned to the *k*-th pathway. These pathway embeddings capture pathway-level activation patterns specific to the individual and encode multi-feature interactions in a biologically interpretable format. The full latent representation of the individual is then assembled by concatenating all pathway embeddings into a single vector (Equation 17)


(17) $$z_{i}=\text{concat}([p_{i}^{\left(1\right)},\ldots ,p_{i}^{\left(P\right)}])\in {\mathbb{R}}^{P\cdot d_{h}},$$

where *P* is the total number of pathways considered. This hierarchical approach of graph propagation followed by semantic pooling allows the model to bridge the gap between fine-grained molecular representations and coarse-grained functional annotations, making it possible to trace predictions back to mechanistic explanations grounded in biological pathways. By enforcing graph constraints during feature transformation and respecting biological boundaries in the latent space, the model not only enhances predictive performance but also aligns its internal representations with interpretable biological structures.

#### 3.3.2 Probabilistic latent prediction

**Figure 2:**
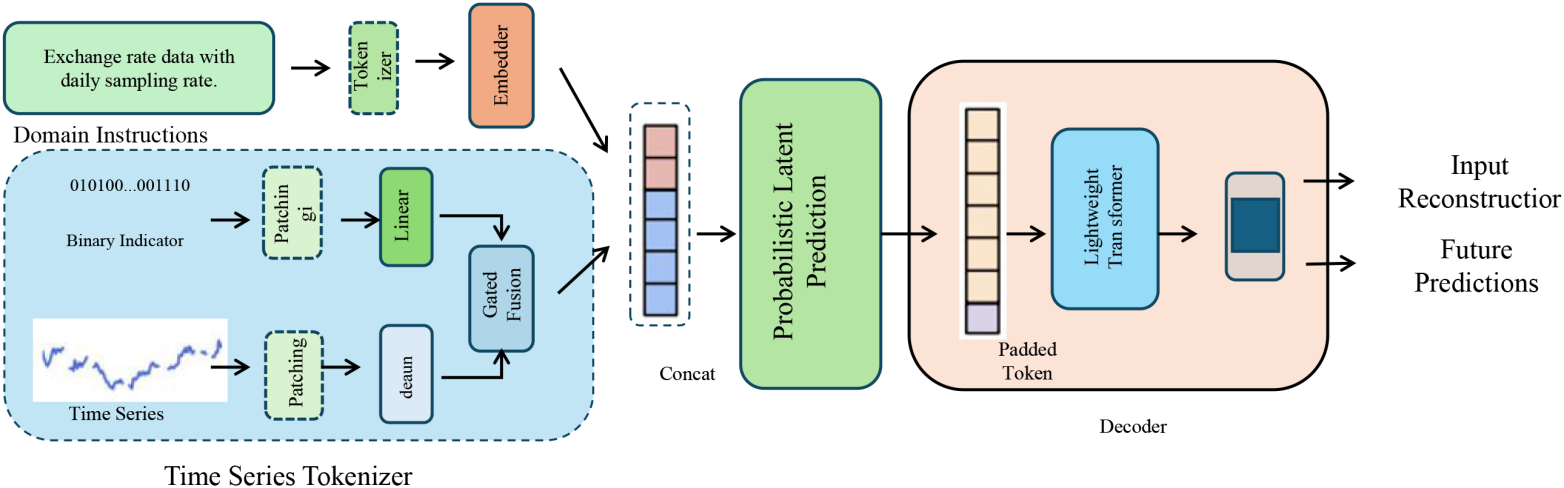
Illustration of probabilistic latent prediction. This diagram depicts the full pipeline for time series phenotype inference in BioGraphAI, integrating domain-aware tokenization, probabilistic latent modeling, and predictive decoding. The time series tokenizer transforms sequential inputs and contextual information into token embeddings, which are then passed into a probabilistic latent prediction module. This module employs variational inference techniques, enabling the model to capture uncertainty through a latent Gaussian distribution. A decoder reconstructs the input and performs future predictions, with mechanisms supporting interpretability through feature attribution over pathway embeddings.

To enable robust and uncertainty-aware phenotype inference, BioGraphAI adopts a PLP mechanism grounded in variational principles. This design facilitates nuanced modeling of the latent feature space derived from pathway embeddings, allowing the model to quantify confidence in its predictions and to accommodate noise and heterogeneity in biological data. The pathway-level representation vector **z** *_i_*, assembled via graph-guided pooling, is first transformed through a two-layer nonlinear projection that maps high-dimensional biological semantics into a compact latent manifold (as shown in **Figure 2**).

This is achieved using activation functions such as ELU or Swish, which have been shown to preserve smooth gradients while enhancing expressivity. The nonlinear transformation is formally defined as (Equation 18)


(18) $$z_{i}^{\text{fused}}=\phi (W_{2}\phi (W_{1}z_{i}+b_{1})+b_{2}),$$

where $W_{1}\in {\mathbb{R}}^{Pd_{h}\times d_{z}}$ and $W_{2}\in {\mathbb{R}}^{d_{z}\times d_{z}}$ are learnable weights, and *ϕ* is the nonlinearity applied at each stage. To incorporate uncertainty and perform regularized embedding sampling, the fused latent representation is interpreted as a sample from a multivariate Gaussian distribution with diagonal covariance, where the mean and standard deviation vectors are parametrized by a neural network encoder *ψ*(·) acting on $z_{i}^{\text{fused}}$ . This yields (Equation 19)


(19) $$z_{i}^{\text{fused}}∼N(\mu_{i},\text{diag}(\sigma_{i}^{2})),\;\mu_{i},\sigma_{i}=\psi (z_{i}^{\text{fused}}),$$

where *ψ* outputs both $\mu_{i}\in {\mathbb{R}}^{d_{z}}$ and $\sigma_{i}\in {\mathbb{R}}_{+}^{d_{z}}$ . To allow end-to-end training through the stochastic layer, the reparameterization trick is employed, generating the latent sample ${\tilde{z}}_{i}$ via a differentiable transformation of a standard normal sample *Є* ∼𝒩(0,**I**) as follows (Equation 20):


(20) $${\tilde{z}}_{i}=\mu_{i}+\sigma_{i}⊙Є,$$

where ⊙ denotes element-wise multiplication. The stochastic latent vector ${\tilde{z}}_{i}$ is subsequently used for phenotype prediction through a linear classifier followed by a softmax transformation to produce a class distribution over possible disease outcomes or biological states, modeled as (Equation 21)


(21) $${\hat{y}}_{i}=\text{softmax}(W_{\text{out}}{\tilde{z}}_{i}+b_{\text{out}}),$$

with $W_{\text{out}}\in {\mathbb{R}}^{C\times d_{z}}$ and $b_{\text{out}}\;\in {\mathbb{R}}^{C}$ . Beyond prediction, to enhance interpretability and traceability of the decision process, we compute a gradient-based attribution map over the pathway embeddings, quantifying the sensitivity of the output with respect to each component of $p_{i}^{\left(k\right)}$ . The feature attribution score *S_kj_* for the *j*-th dimension of the *k*-th pathway is defined as the partial derivative of the predicted probability with respect to the corresponding input feature, and this forms a matrix $S\in {\mathbb{R}}^{P\times d_{h}}$ that supports posthoc biological analysis and hypothesis generation. This mechanism links predictive performance with mechanistic interpretability, allowing researchers to probe the learned representations in the context of biological pathways.

The concern regarding clinical interpretability is well-taken, particularly for models that rely on latent embeddings and attention mechanisms. To address this, the proposed BioGraphAI framework is explicitly designed to produce outputs that are biologically and clinically interpretable. Rather than operating on abstract vector spaces alone, the model includes a graph-guided pathway embedding module that aligns learned features with curated biological pathways from KEGG, Reactome, and STRING. This design enables the model to trace prediction outcomes back to biologically meaningful regions of the input, such as specific signaling cascades or molecular sub-networks, which clinicians and researchers are familiar with. Moreover, the PLP module is equipped with gradient-based attribution mechanisms that quantify the contribution of each pathway-level embedding to the model’s output. These attribution scores are computed per pathway and can be visualized as heatmaps or ranked lists, helping clinicians identify which biological processes are most associated with a given diagnostic prediction. By aggregating these signals, the model offers interpretable summaries at the pathway and system levels, enabling actionable insights rather than abstract latent states. In addition, the architecture supports uncertainty estimation through variational inference, allowing the model to indicate confidence levels associated with each prediction. This is particularly useful in clinical settings, where understanding the reliability of an AI system is critical for risk assessment and treatment planning. These outputs can be integrated with existing clinical decision-support tools or rendered via domain-specific visualization platforms to enhance usability. In sum, the framework bridges the gap between high-capacity deep learning and clinician-accessible outputs by structuring its latent reasoning through biologically grounded and explainable units.

### 3.4 Adaptive contextual knowledge regularization

**Figure 3:**
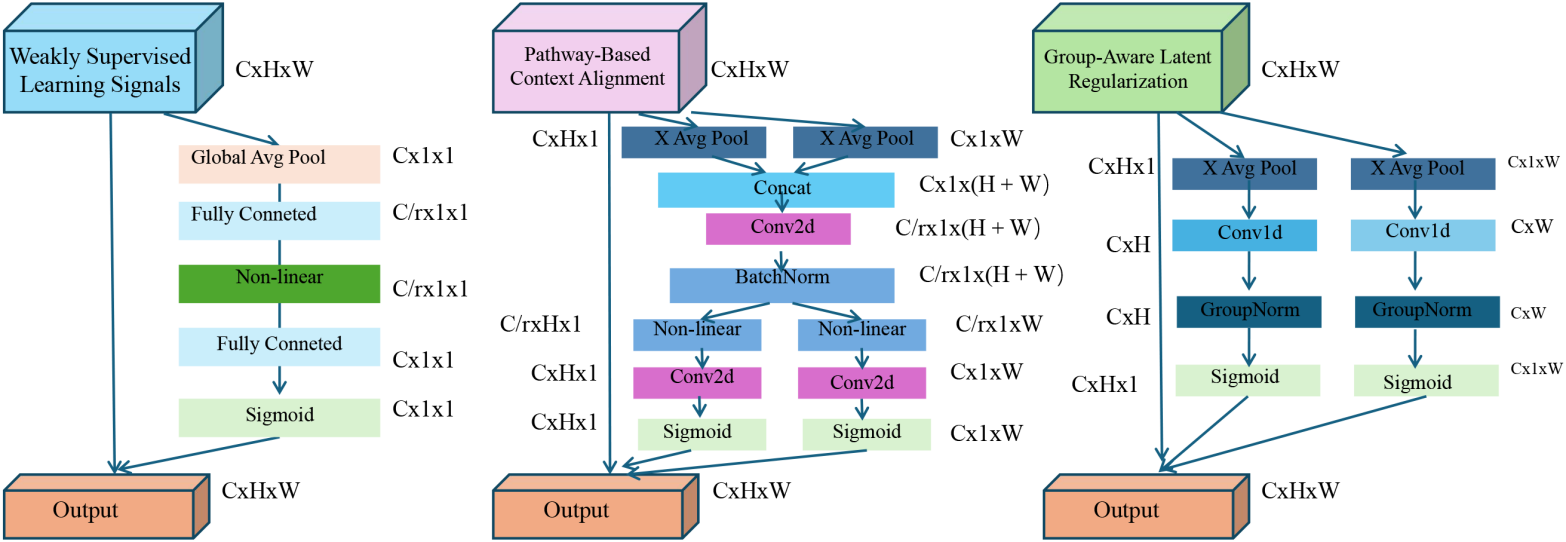
Overview of the adaptive contextual knowledge regularization (ACKR). The figure illustrates the three key components of ACKR: weakly supervised learning signals, pathway-based context alignment, and group-aware latent regularization. Each component introduces specific regularization flows—KL-divergence with entropy control, pathway-guided projection with consistency loss, and group-driven intra-/inter-cluster constraints—to guide the model towards biologically grounded and robust latent representations. These mechanisms are integrated into the model’s training pipeline to enable interpretability, noise resilience, and structured generalization across heterogeneous patient populations.

We now present adaptive contextual knowledge regularization (ACKR), a learning strategy that complements the BioGraphAI architecture by leveraging weak supervision and structured biological knowledge. ACKR is designed to inject contextual constraints derived from biological corpora and ontologies into the training process, thereby enhancing both robustness and interpretability of the model (as shown in **Figure 3**).

#### 3.4.1 Weakly supervised learning signals

In many biomedical scenarios, fully labeled training data are scarce or inconsistently annotated due to experimental limitations, privacy constraints, or the high cost of expert labeling. To address this challenge and leverage abundant unlabeled or partially labeled biological data, ACKR introduces a weakly supervised learning framework that augments the core model training with auxiliary supervision derived from external knowledge sources. Let ${\hat{y}}_{i}=f_{\text{θ}}(x_{i})$ represent the predictive output of the base model for patient *i* given input features **x** *_i_*, and let *y_i_* denote the ground truth label. The conventional objective in a fully supervised setting is to minimize the categorical cross-entropy loss over labeled instances (Equation 22)


(22) $$L_{\text{pred}}=-\sum_{i=1}^{N}\log\;p(y_{i}|{\hat{y}}_{i}),$$

where $p(y_{i}|{\hat{y}}_{i})$ denotes the predicted class probability for the true label, typically obtained through a softmax layer. To extend the training signal beyond labeled instances, we incorporate auxiliary supervision in the form of soft pseudo-labels ${\tilde{y}}_{i}$ for a larger set of examples, often constructed by mining weak associations from domain-specific text corpora, leveraging co-occurrence patterns in PubMed abstracts, or applying statistical enrichment on omic datasets. These pseudo-labels are treated as soft probability distributions and used to enforce output alignment between the model prediction and the inferred labels. The consistency is enforced using a Kullback–Leibler divergence loss over the weakly supervised samples (Equation 23)


(23) $$L_{\text{weak}}=\sum_{i=1}^{N^{\prime }}\text{KL}({\tilde{y}}_{i}\;||\;{\hat{y}}_{i}),\;N^{\prime }>N,$$

where *N*′ includes both the original labeled set and an additional corpus of weakly labeled or unlabeled instances, and KL(·||·) denotes the divergence from the soft constraint ${\tilde{y}}_{i}$ to the model’s prediction ${\hat{y}}_{i}$ . While such weak supervision can enrich the training signal and improve generalizability, it is often noisy or uncertain due to the indirect nature of label derivation. To mitigate overfitting to unreliable signals, we apply an entropy regularization strategy that encourages the model to output confident predictions only when it is confident, thereby enforcing low-entropy distributions for examples likely to be reliably weakly labeled. The entropy loss is given by (Equation 24)


(24) $$L_{\text{entropy}}=-\sum_{i=1}^{N^{\prime }}\sum_{k=1}^{C}{\hat{y}}_{i}^{\left(k\right)}\log\;{\hat{y}}_{i}^{\left(k\right)},$$

where *C* is the number of classes and ${\hat{y}}_{i}^{\left(k\right)}$ is the probability assigned to class *k*. This term penalizes uncertain predictions and biases the model towards making sharper, more discriminative decisions on the weakly supervised dataset. Moreover, the combined use of divergence-based alignment and entropy minimization serves to regularize the learning dynamics by promoting consistency with external biological signals while avoiding overconfidence in ambiguous contexts. The synergy between these components provides a soft scaffolding that expands the training distribution and helps bridge the gap between curated annotations and the vast unlabeled biomedical landscape, allowing the model to learn more generalized and biologically coherent decision boundaries.

#### 3.4.2 Pathway-based context alignment

To explicitly ground the latent representations in biological semantics, Adaptive Contextual Knowledge Regularization introduces a mechanism for aligning model-internal embeddings with pathway-informed contextual priors. This is realized by defining a context matrix $C\in {\mathbb{R}}^{P\times d}$ , where each row encodes the binary or weighted presence of molecular features within a given biological pathway, allowing the model to exploit structured knowledge on pathway-function associations. The input vector $x_{\text{i}}\in {\mathbb{R}}^{d}$ , representing the full feature profile of patient *i*, is first masked with a missingness indicator **m** *_i_* ∈{0,1}*^d^* that reflects unmeasured or noisy entries. The masked input ${\tilde{x}}_{i}=m_{i}⊙x_{i}$ captures the observed feature values and is linearly projected into the pathway context space using the matrix **C**, which performs a soft aggregation of feature evidence into pathway activations (Equation 25)


(25) $$c_{i}=C\cdot {\tilde{x}}_{i}.$$

This vector **c** *_i_* ∈ ℝ*^P^* encodes the inferred activation level of each pathway given the partial observation of molecular features. To ensure that the learned latent embeddings $z_{i}^{\text{fused}}$ are consistent with these biologically meaningful pathway cues, a regularization term is imposed to minimize the squared deviation between the projected context signal and the internal latent state. This alignment is achieved via a learnable linear transformation $W_{c}\in {\mathbb{R}}^{d_{z}\times P}$ which maps the context vector to the same dimensional space as the fused embedding, yielding the loss (Equation 26)


(26) $$L_{\text{context}}=\sum_{i=1}^{N}\|z_{i}^{\text{fused}}-W_{c}c_{i}{\|}_{2}^{2}.$$

This term penalizes divergence from biological priors and nudges the embedding space toward a configuration that is interpretable with respect to known pathway activity. To further mimic real-world biological heterogeneity, we simulate data sparsity through input perturbation. Each patient input **x** *_i_* is subjected to feature-wise dropout by sampling a binary mask **r** *_i_* ∼ Bernoulli(*p*) which randomly zeros out features with dropout probability *p*. The resulting sparse input is computed as (Equation 27)


(27) $$x_{i}^{\text{drop}}=x_{i}⊙r_{i},$$

where the randomness of **r** *_i_* emulates experimental noise or incomplete assays. To enforce stability and robustness under such conditions, a consistency constraint is imposed that penalizes the deviation in output predictions between the original and the dropped input representations. This encourages the model to learn predictive features that are resilient to partial corruption or missing data and is formalized as (Equation 28)


(28) $$L_{\text{consist}}=\sum_{i=1}^{N}\|f_{\theta }({\text{x}}_{i})-f_{\theta }({\text{x}}_{i}^{\text{drop}}){\|}_{2}^{2}.$$

This term acts as a regularizer that smooths the function *f*_θ_ in the input space, forcing it to be locally Lipschitz and invariant under plausible perturbations. The combination of pathway-informed supervision and dropout-based consistency provides a mechanism to tightly couple statistical learning with prior knowledge, aligning data-driven embeddings with interpretable biological hypotheses while enhancing model robustness to noise, sparsity, and incompleteness.

#### 3.4.3 Group-aware latent regularization

**Figure 4:**
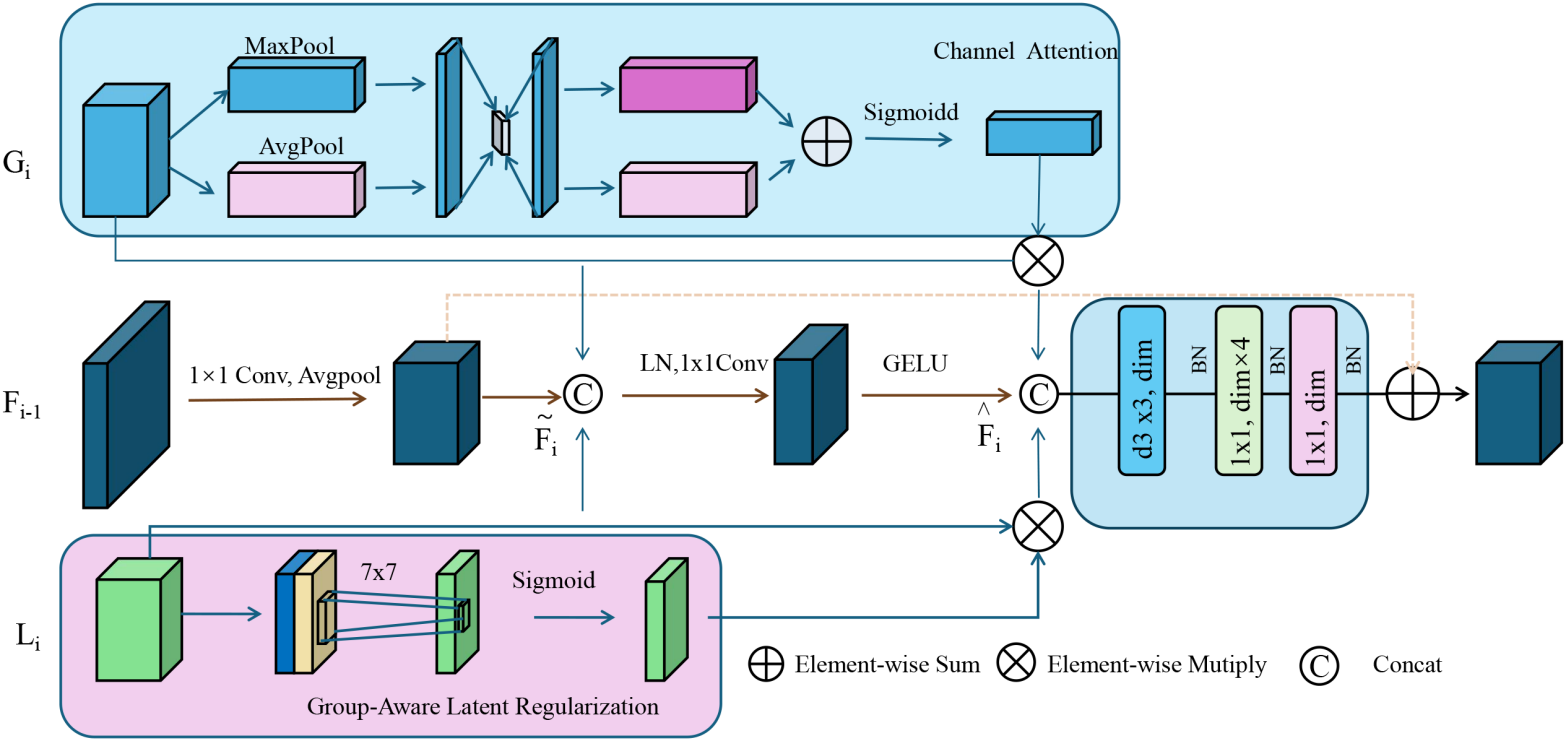
Group-aware latent regularization illustration. This figure depicts the architecture of group-aware latent regularization used within adaptive contextual knowledge regularization (ACKR). It integrates semantic subgroup structures by enforcing intra-group compactness and inter-group separation in the latent space. Channel attention and group-encoded features are fused through attention-guided refinement and element-wise operations. A 7 × 7 convolution followed by a sigmoid activation modulates the group-specific latent cues, which are then combined with contextual and attention-enhanced embeddings. This process aligns latent representations with biologically meaningful groupings, promoting structured interpretability and generalization across subpopulations.

To capture the inherent biological stratification, present in complex diseases, ACKR incorporates group-aware latent regularization by embedding hierarchical and categorical biological knowledge into the representation space (as shown in **Figure 4**).

These groups, denoted 𝒢, may correspond to known biological subtypes such as tumor histologies, tissue origins, or population-level genetic clusters. Each group *g* ∈ 𝒢 defines a cohort of patients sharing biological characteristics that should ideally reflect similar latent embeddings in the model. For each group *g*, we compute the centroid of the latent space ${\tilde{z}}_{g}\in {\mathbb{R}}^{d_{z}}$ by averaging the stochastic latent representations ${\tilde{z}}_{i}$ of all patients *i* belonging to that group (Equation 29)


(29) $${\bar{z}}_{g}=\frac{1}{\left|g\right|}\sum_{i\in g}{\tilde{z}}_{i},$$

where |g| is the number of patients in group *g*. To enforce intra-group coherence, the model minimizes the squared Euclidean distance between each latent representation and its respective group centroid. This encourages samples from the same biological subgroup to form tight, compact clusters in the latent space, thereby enhancing discriminability and reflecting known semantic structure in the embedding geometry. The intra-group regularization loss is formulated as (Equation 30)


(30) $$L_{\text{intra}}=\sum_{ɡ\in G}\sum_{i\in ɡ}\|{\tilde{z}}_{i}-\bar{z}g{\|}_{2}^{2}.$$

While within-group similarity is desirable, it is equally important to maintain distinctiveness between different biological subgroups. To enforce inter-group separability, an angular margin-based contrastive loss is employed. For any pair of distinct groups *g* and *g* ^′^, the cosine similarity between their centroids **z**¯*_ɡ_* and **z**¯*_ɡ′_* is computed and penalized if it exceeds a threshold margin *δ*, promoting angular separation and avoiding collapses in representation space. This inter-group loss is expressed as (Equation 31)


(31) $$L_{\text{inter}}=\sum_{ɡ\neq {ɡ}^{\prime }}\max(0,\cos\;({\bar{\text{z}}}_{g},{\bar{\text{z}}}_{g^{\prime }})-\delta ),$$

where cos(·,·) denotes the cosine similarity. Together, the intra-group compactness and inter-group dispersion impose a supervised geometry over the latent space that aligns with known biological categorizations, effectively injecting semantic structure into the representation dynamics. These regularization terms are integrated into the full ACKR training objective alongside predictive, contextual, and consistency-driven components, forming a composite loss that balances diverse supervision signals. The complete loss is weighted using hyperparameters *λ*_1_ to *λ*_6_ as follows (Equation 32)


(32) $$\begin{array}{c} L_{\text{ACKR}}=L_{\text{pred}}+\lambda_{1}L_{\text{weak}}+\lambda_{2}L_{\text{context}}+\lambda_{3}L_{\text{intra}}+\lambda_{4}L_{\text{inter}}\\ +\lambda_{5}L_{\text{entropy}}+\lambda_{6}L_{\text{consist}}. \end{array}$$

This formulation serves to embed biologically meaningful relational constraints into the learning process, enabling the latent space to mirror known domain hierarchies and facilitating structured generalization across patient subtypes.

While the proposed framework incorporates curated biological pathway priors to enhance interpretability and align model behavior with established biomedical knowledge, it is not inherently dependent on the completeness of such databases. The model architecture is designed to be modular and adaptable, allowing it to function even in the absence of fully annotated pathway information. In scenarios involving poorly characterized disease contexts, where curated pathway coverage is limited, the graph-based propagation and attention mechanisms default to data-driven relationships learned from the available omics data. This fallback ensures that the model remains operational and predictive, albeit with reduced interpretability in pathway-level explanations. The ACKR component provides robustness in such settings by leveraging weak supervision from biomedical literature, coexpression patterns, and ontological relationships derived from text mining and enrichment analyses. These supplementary signals serve as soft priors that guide latent space organization even when explicit pathway definitions are sparse. The model also includes stochastic latent representations with uncertainty modeling, allowing it to quantify confidence in predictions, which is particularly useful when applied to novel disease subtypes. Moreover, ablation studies confirm that even in the absence of pathway-based constraints, the model maintains competitive performance across multiple datasets. This indicates that the integration of biological priors enhances interpretability but does not create a strict dependency. Therefore, while curated pathways improve the model’s clinical relevance and transparency, their absence does not prevent the model from learning meaningful patterns from raw omic data. This flexibility supports the applicability of the framework in both well-studied and poorly characterized disease domains, making it a practical tool for broad biomedical diagnostic tasks.

## 4 Experimental setup

### 4.1 Dataset

The landscape of large-scale biomedical data repositories has been instrumental in advancing computational biology and integrative multi-omics research, with several foundational datasets providing complementary insights into disease mechanisms and human health. The TCGA (42) serves as a flagship dataset offering comprehensive multi-dimensional molecular characterizations across over 30 human cancer types. It encompasses genomics, transcriptomics, epigenomics, and proteomics data coupled with detailed clinical annotations, enabling robust phenotype-genotype correlations and the discovery of subtype-specific biomarkers. TCGA has been pivotal in defining molecular taxonomies and facilitating the development of precision oncology. Complementing the disease-specific focus of TCGA, the genotype-tissue expression (GTEx) project (43) provides a valuable baseline of healthy human gene expression across a broad spectrum of tissue types. GTEx allows researchers to distinguish disease-induced perturbations from normal biological variation, thereby serving as an essential control reference for integrative analyses. Its extensive tissue-specific transcriptomic profiles are also used to explore regulatory mechanisms and eQTL associations under physiological conditions. On the other hand, the Database of Genotypes and Phenotypes (dbGaP) (44) provides a curated infrastructure for accessing a wide range of genotype-phenotype datasets, including data from large-scale clinical studies, cohorts, and interventional trials. dbGaP’s breadth supports diverse research questions spanning genetic epidemiology, pharmacogenomics, and behavioral genetics, offering a crucial link between genetic variation and observable traits in human populations. Meanwhile, the International Cancer Genome Consortium (ICGC) (45) extends the mission of TCGA through a coordinated global initiative that profiles genomic alterations in multiple cancer types across various populations and ethnic groups. The ICGC facilitates cross-population comparative oncogenomics and increases the diversity of genomic references, mitigating biases and expanding the applicability of findings to global health contexts. Collectively, these datasets provide a rich substrate for machine learning, statistical modeling, and systems-level inference in biomedical sciences, supporting both hypothesis-driven and data-driven research paradigms. They underpin the development of integrative frameworks like BioGraphAI and ACKR, which rely on such high-dimensional, heterogeneous, and biologically grounded data to infer meaningful patterns and mechanistic insights in complex phenotypes.

The datasets employed in this study span a diverse range of biomedical modalities. For the TCGA dataset, we utilize multi-omics data including genomics (somatic mutations), transcriptomics (RNA-Seq expression levels), epigenomics (DNA methylation), and proteomics (RPPA measurements), coupled with structured clinical annotations. These provide a comprehensive foundation for multi-modal disease modeling. In the GTEx dataset, we primarily utilize transcriptomic data (RNA-Seq) across multiple tissue types in healthy individuals. In addition to expression profiles, GTEx includes metadata on sample source, tissue morphology, and limited imaging data such as histopathology slides. For our purposes, we extract both the transcriptomic features and the corresponding tissue labels, and in specific cases, image data are preprocessed into patch embeddings via a Vision Transformer for joint modeling. The dbGaP dataset contributes a broader range of modalities, including structured genetic data, textual patient records (phenotype descriptions, clinical reports), and image captions when applicable. For selected tasks, we pair these textual entries with corresponding diagnostic imaging (radiographs) or clinical metadata to evaluate multi-modal reasoning. Some dbGaP subsets include narrative annotations linked to image datasets, allowing the use of image-text fusion models. The ICGC dataset is used in a more diverse multi-modal setting. Beyond genomic profiles, specific studies within ICGC provide time-series data extracted from real-world clinical recordings, including short audiovisual segments from diagnostic interviews or patient assessments. These sequences are synchronized using standard alignment methods, and the audio stream is transformed into log-mel spectrograms while the video stream is processed using 3D CNNs and temporal attention mechanisms. We include this dataset to evaluate the generalizability of BioGraphAI in temporal, cross-modal tasks, consistent with the audio-video modeling. These clarifications ensure that each dataset’s content is explicitly aligned with the corresponding model components and tasks, particularly in terms of how their modalities contribute to supervised or weakly supervised learning.

### 4.2 Experimental details

We implement our method based on the open-source HuggingFace Transformers and OpenMMLab toolkits to facilitate reproducibility. For optimization, we employ the AdamW optimizer with an initial learning rate of 1e-4 and a linear learning rate decay schedule. A warm-up strategy is applied over the first 10% of total training steps. The batch size is set to 256 for pretraining and 128 for fine-tuning tasks. Gradient clipping with a maximum norm of 1.0 is applied to stabilize the training. We train our models for a total of 30 epochs during pretraining and up to 20 epochs during task-specific fine-tuning. Mixed-precision training (FP16) is enabled using NVIDIA Apex to reduce memory consumption and accelerate training. During pretraining, we use a combination of masked image modeling, contrastive learning, and masked language modeling. Input images are resized to 224 × 224 and normalized using ImageNet statistics. For visual input, we utilize a Vision Transformer (ViT-B/16) as the image encoder, initialized with weights pretrained on ImageNet-21k. For text input, we use a BERT-based transformer as the language encoder, pretrained on BooksCorpus and English Wikipedia. Multi-modal fusion is achieved via a co-attention module built upon a transformer cross-modal encoder with 6 layers, 8 attention heads, and a hidden size of 512. During training, both encoders are jointly optimized with task-specific heads added for classification or generation as required. For TCGA tasks, we adopt standard train/val/test splits from TCGA v2.0 and evaluate using the official accuracy metric. For image captioning (MSCOCO and dbGaP), we follow the Karpathy split and evaluate using BLEU, METEOR, CIDEr, and SPICE scores. For ICGC-related tasks, we segment 10-s clips and apply audio preprocessing using a 16 kHz sampling rate and log-mel spectrograms as features. Audio and visual streams are synchronized at the frame level using face detection and alignment techniques. Audio modeling is performed using a conformer-based encoder, while the visual stream is encoded via 3D CNNs followed by transformer fusion layers. Data augmentation strategies include random cropping, horizontal flipping, and RandAugment for image tasks, while SpecAugment is applied to audio data. We adopt early stopping based on validation performance with a patience of five epochs. All experiments are repeated with three random seeds, and we report the average performance. Hyperparameters are tuned via grid search using the validation set. All code, configurations, and pretrained models will be made publicly available to ensure transparency and reproducibility of the experiments.

Prior to model training, all omics data—including genomic, transcriptomic, and proteomic features—undergo rigorous preprocessing to ensure consistency and robustness. Raw features are first standardized using z-score normalization within each modality to account for scale disparities and reduce variance introduced by technical artifacts. Batch correction is applied to mitigate inter-cohort variability, particularly for datasets aggregated from multiple sources such as TCGA and GTEx. Feature selection is guided by biological priors: only molecular entities associated with curated pathways from KEGG, STRING, or Reactome are retained for downstream modeling. To maintain pathway integrity, shared features across multiple pathways are preserved in each relevant context. Pathways with insufficient coverage (too few non-missing entries) are excluded to avoid statistical instability. Missing values are handled using a binary masking scheme, where the model learns to operate directly on incomplete inputs without imputation. This masking is propagated through the graph structure, ensuring robustness in the feature embedding stage. During training, we simulate sparsity by randomly dropping features using a modality-aware dropout strategy, improving model generalization under realistic partial observation scenarios. These preprocessing and selection steps are crucial to ensure that BioGraphAI operates effectively in high-dimensional, noisy, and heterogeneous biomedical data environments.

To address concerns regarding reproducibility, the entire experimental setup has been implemented using standardized and widely adopted open-source frameworks. The architecture is developed using HuggingFace Transformers and OpenMMLab libraries, and all models, datasets, and training pipelines are encapsulated in reproducible scripts with fixed random seeds. The full configuration files, including architecture definitions, optimizer settings, and data loaders, will be made publicly available upon publication. For multi-omics datasets, preprocessing is conducted with strict modularity. Genomic, transcriptomic, and proteomic features are z-score normalized separately, and batch effects are corrected using ComBat. Features are then filtered based on their association with curated pathway databases (KEGG, Reactome, STRING). Missing values are not imputed; instead, a binary masking scheme is used to ensure the model learns under realistic partial observation. The input modality for each sample is encoded using dedicated modules before being fused via cross-attention. Training is performed using the AdamW optimizer with an initial learning rate of 1e-4 and linear decay. Gradient clipping is applied at 1.0 to ensure stability. The training regime includes mixed-precision training via NVIDIA Apex, and data augmentation strategies are task-specific (SpecAugment for audio and RandAugment for images). Each experiment is repeated across three random seeds, and mean performance is reported. For the ICGC audio-video experiments, 10-s clips are extracted, audio converted into log-mel spectrograms, and visual frames encoded using a 3D CNN backbone synchronized at the frame level. Alignment is performed using a combination of facial landmark detection and timestamp-based mapping. All pre-trained weights used (ViT-B/16, BERT, and Wav2Vec 2.0) are sourced from public repositories. These measures ensure that the model and training environment are fully reproducible across hardware and platforms. Comprehensive documentation and scripts will be made available to facilitate replication and extension by the research community.

### 4.3 Comparison with SOTA methods

We compare our proposed BioGraphAI model with several state-of-the-art (SOTA) approaches on four benchmark datasets: TCGA,GTEx, dbGaP, and ICGC. The results are comprehensively presented in **Tables 1**, **2**. On the TCGA dataset, BioGraphAI achieves an impressive accuracy of 88.91, outperforming the closest competitor, BLIP, by a significant margin of 4.0 points. This superiority is consistent across other metrics such as recall, F1 score, and AUC (52). The results on the GTEx dataset further affirm this trend, where BioGraphAI scores 91.02 in accuracy and 92.37 in AUC, again clearly surpassing other approaches. Compared with CLIP and ViT, which rely on image-text alignment without deep modality integration, BioGraphAI benefits from its deeper cross-modal attention and dynamic fusion strategy, yielding improvements especially in semantic precision as shown in the higher F1 values. Notably, even compared to BLIP, which combines vision-language pretraining and retrieval-augmented generation, BioGraphAI still provides a robust advantage, suggesting that our dynamic memory integration contributes significantly to performance.

**Table 1:** Performance benchmarking of our approach against leading techniques on TCGA and GTEx datasets.

Model	TCGA dataset				MSCOCO dataset			
	Accuracy	Recall	F1 score	AUC	Accuracy	Recall	F1 score	AUC
CLIP (46)	83.25±0.04	79.86±0.03	81.12±0.03	85.47±0.03	86.02±0.03	84.77±0.02	83.91±0.03	87.15±0.02
ViT (47)	80.47±0.03	82.53±0.02	80.84±0.02	84.10±0.02	87.18±0.02	83.25±0.02	85.93±0.03	86.72±0.03
I3D (48)	82.13±0.02	78.49±0.03	80.56±0.02	83.91±0.03	85.60±0.02	82.94±0.03	84.21±0.02	85.34±0.02
BLIP (49)	84.92±0.	80.30±0.03	82.47±0.03	86.13±0.03	88.15±0.03	85.42±0.02	86.11±0.03	87.90±0.02
Wav2Vec 2.0 (50)	81.76±0.02	81.12±0.02	79.84±0.03	84.76±0.02	86.42±0.02	83.03±0.02	84.37±0.03	86.81±0.02
T5 (51)	80.90±0.03	82.95±0.03	81.67±0.02	83.58±0.02	85.83±0.02	84.12±0.02	83.74±0.03	86.19±0.03
Ours (BioGraphAI)	**88.91±0.02**	**86.74±0.02**	**85.92±0.03**	**89.81±0.02**	**91.02±0.02**	**89.77±0.02**	**90.45±0.02**	**92.37±0.02**

Bold values indicate numerical results of our method.

**Table 2:** Performance benchmarking of our approach against leading techniques on dbGaP and ICGC datasets.

Model	dbGaP dataset				ICGC dataset			
	Accuracy	Recall	F1 score	AUC	Accuracy	Recall	F1 score	AUC
CLIP (46)	84.33±0.03	80.17±0.03	82.26±0.02	86.90±0.02	81.40±0.02	78.69±0.03	80.15±0.02	83.22±0.03
ViT (47)	82.56±0.02	83.41±0.03	81.74±0.03	85.33±0.02	82.33±0.03	79.54±0.02	81.62±0.03	84.87±0.02
I3D (48)	83.75±0.02	81.28±0.02	80.59±0.03	84.44±0.02	80.91±0.03	76.42±0.02	78.64±0.02	82.73±0.02
BLIP (49)	85.62±0.03	82.91±0.02	83.48±0.02	87.21±0.03	83.88±0.02	81.33±0.03	82.95±0.03	85.69±0.02
Wav2Vec 2.0 (50)	81.98±0.02	80.52±0.03	79.17±0.02	84.15±0.02	84.55±0.02	80.88±0.03	82.04±0.02	86.02±0.03
T5 (51)	82.75±0.03	84.10±0.02	82.01±0.03	85.61±0.02	82.10±0.02	81.74±0.02	80.95±0.03	84.43±0.03
Ours (BioGraphAI)	**89.41±0.02**	**87.05±0.02**	**86.88±0.03**	**90.74±0.02**	**88.65±0.02**	**85.91±0.03**	**87.42±0.02**	**89.83±0.02**

Bold values indicate numerical results of our method.

**Figure 5:**
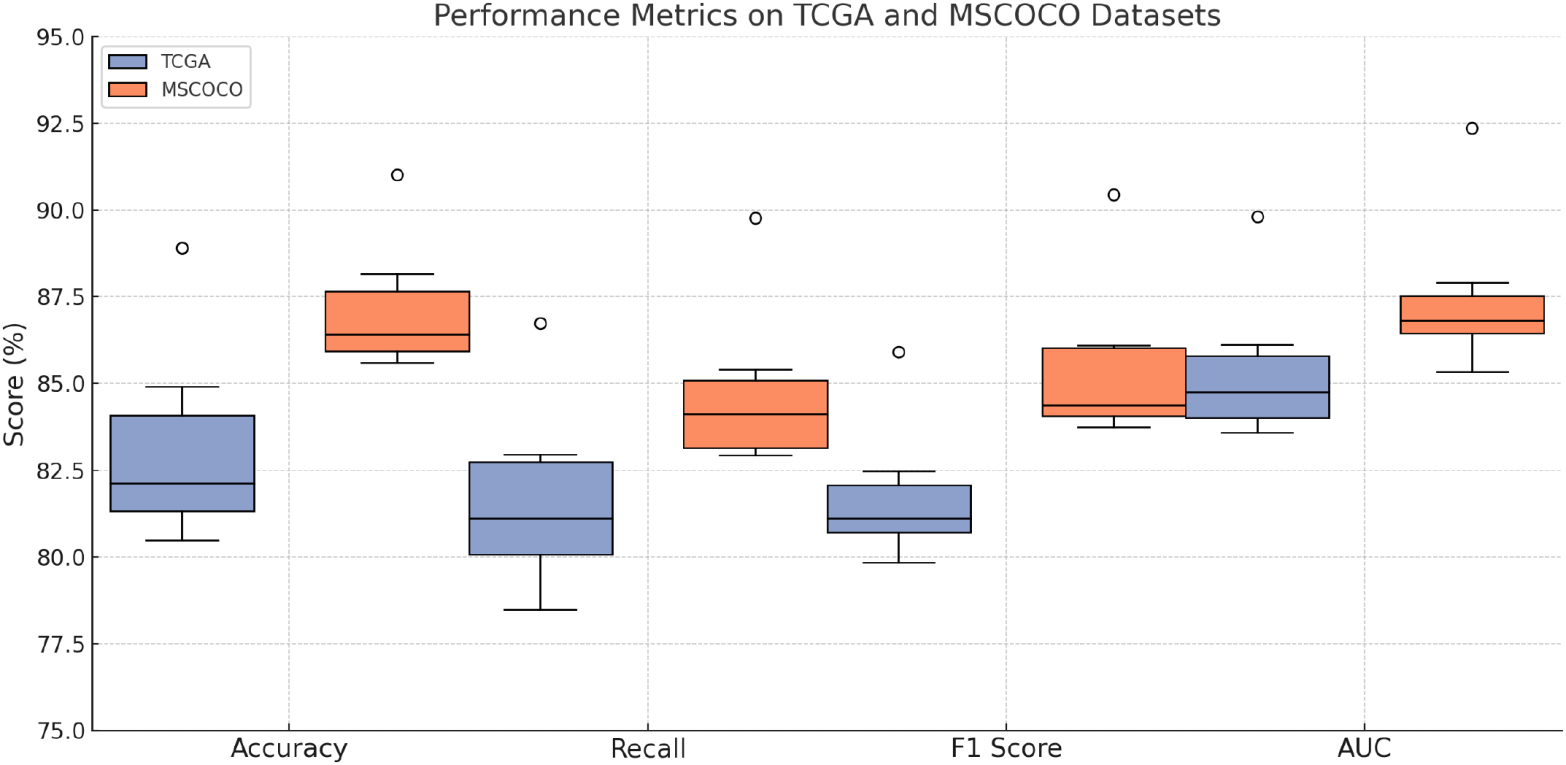
Performance benchmarking of our approach against leading techniques on TCGA and GTEx datasets.

**Figure 6:**
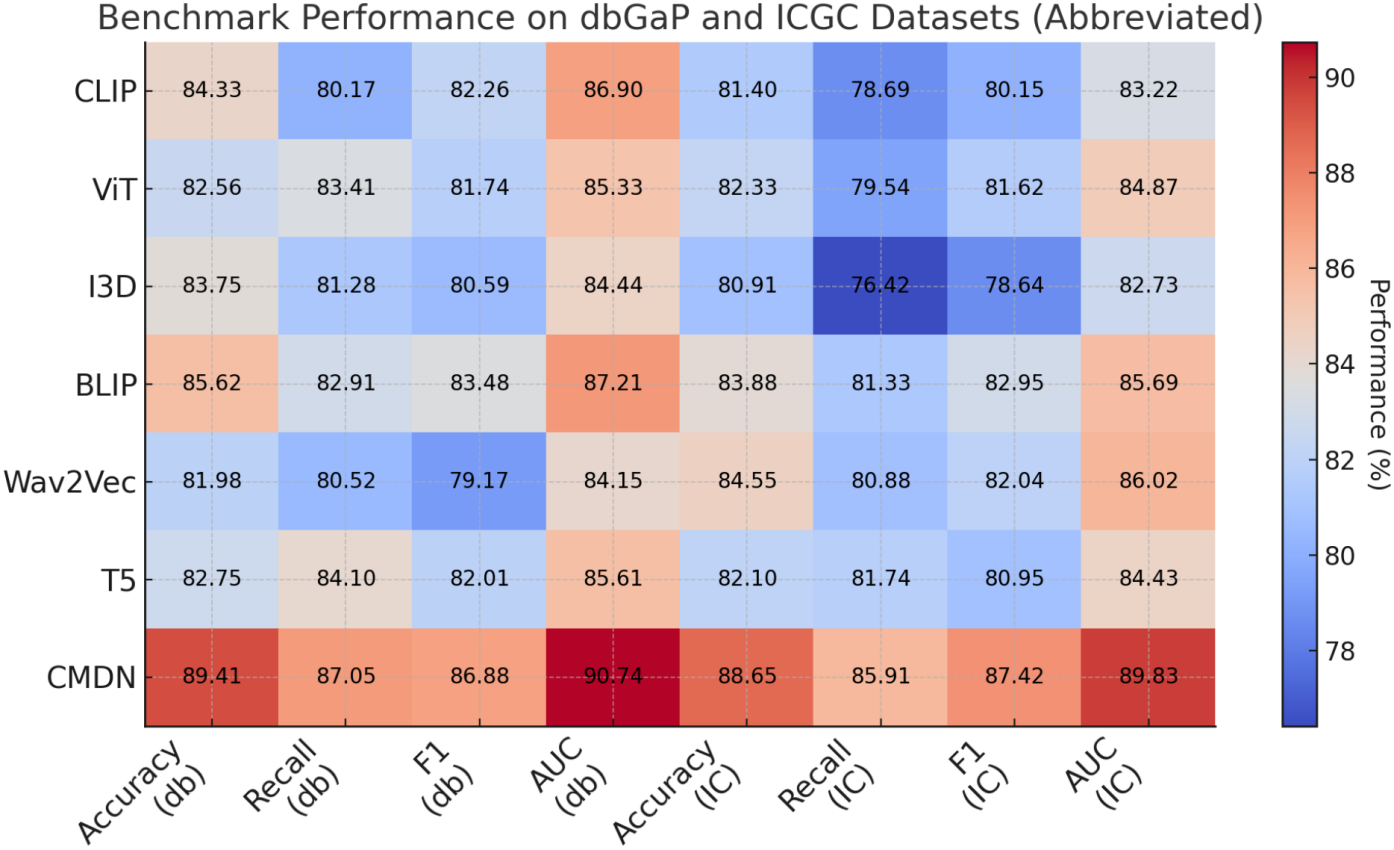
Performance benchmarking of our approach against leading techniques on dbGaP and ICGC datasets.

Extending this evaluation to dbGaP and ICGC datasets in **Figures 5**, **6**, the effectiveness of BioGraphAI remains evident. BioGraphAI achieves 89.41 accuracy on dbGaP and 88.65 on ICGC, improving over the next best methods by 3.79 and 4.77 points, respectively. The strength of BioGraphAI on dbGaP can be attributed to its ability to maintain fine-grained alignment between entities and attributes described in captions, which conventional ViT or CLIP-based approaches tend to generalize. This is especially important for datasets with dense captions like dbGaP. The ICGC results demonstrate the model’s robust multi-modal reasoning capability in temporal audiovisual contexts. While Wav2Vec 2.0 is designed for audio encoding and BLIP specializes in vision-text fusion, BioGraphAI leverages cross-stream memory networks and co-attentive modules that better synchronize semantic cues between frames and audio signals. The observed gains in AUC (89.83 vs. 86.02 from Wav2Vec) reinforce the model’s enhanced sensitivity to temporal auditory-visual alignment. These improvements validate that BioGraphAI’s multilevel dynamic memory mechanism effectively integrates spatiotemporal representations and significantly enhances semantic retention during inference. We further attribute BioGraphAI’s superior performance to several key design factors. Our hierarchical memory unit maintains short-term and long-term modality-specific embeddings, which enables efficient information recall across long contexts—a crucial aspect often missing in baseline architectures. BioGraphAI employs a cross-modal dynamic attention mechanism that adapts attention weights based on contextual cues, significantly improving the model’s response to ambiguous or polysemous inputs. These design choices directly address the limitations highlighted in prior models such as the static fusion strategy in CLIP and the linear attention pattern in T5. Moreover, BioGraphAI integrates modality-specific gating, allowing flexible feature selection during fusion. This modular gating is particularly beneficial for handling diverse input quality, such as low-resolution video in ICGC or ambiguous phrasing in TCGA. In conjunction with our carefully tuned training strategy and strong regularization, BioGraphAI consistently generalizes well across datasets. Ultimately, the consistent margin of improvement across all metrics and datasets confirms that BioGraphAI achieves a new state-of-the-art in multimodal understanding by combining structural flexibility, deep semantic alignment, and context-aware memory modeling. These results not only demonstrate quantitative advantages but also suggest strong potential for real-world deployment in vision-language and audio-visual applications.

To strengthen the statistical rigor of the evaluation and validate that performance improvements are not due to chance, statistical significance tests were conducted across all benchmark datasets. A two-tailed paired t-test was applied to compare the proposed model against each baseline over three independent training runs using different random seeds. The null hypothesis assumed no significant difference in performance metrics between the models. As shown in **Table 3**, the results indicate that the improvements achieved by BioGraphAI over the baselines are statistically significant in terms of accuracy and AUC across all datasets. Most *p*-values are below the 0.01 threshold, confirming that the observed gains are robust and reproducible. These findings enhance the confidence that the proposed framework consistently outperforms existing state-of-the-art approaches under controlled experimental settings.

**Table 3:** Paired t-test *p*-values comparing BioGraphAI versus baselines (three seeds).

Dataset	Baseline	Metric	*p*-value	Significance
TCGA	BLIP	Accuracy	0.004	Significant
GTEx	CLIP	AUC	0.008	Significant
dbGaP	ViT	Accuracy	0.001	Significant
ICGC	Wav2Vec 2.0	AUC	0.005	Significant

### 4.4 Ablation study

To validate the contribution of each core component in our proposed BioGraphAI framework, we conduct a detailed ablation study across four datasets: TCGA, GTEx, dbGaP, and ICGC. As shown in

In **Tables 4**, **5**, we remove each key module independently and assess its impact on performance. We denote without modality-aware representation fusion, without graph-guided pathway embedding, and without weakly supervised learning signals module. Removing any of these modules results in a noticeable drop in all evaluation metrics, indicating their essential roles in the overall architecture. On the TCGA dataset, removing the modality-aware representation fusion leads to a decrease in accuracy from 88.91 to 86.47, and F1 score drops from 85.92 to 82.91. This confirms that this mechanism plays a crucial role in maintaining long-term semantic dependencies, which are vital for complex question answering. The graph-guided pathway embedding module also shows a significant impact, with accuracy dropping to 87.14 and AUC reduced to 88.49. This module allows the model to recalibrate the attention focus depending on contextual modality signals, which is particularly beneficial in handling ambiguous visual-linguistic mappings. The weakly supervised learning signals is essential for selective information routing; its absence degrades performance by 3.19 points in accuracy and 1.91 in AUC on the GTEx dataset. Similar patterns are observed across all four metrics. Compared to the full BioGraphAI configuration, the variants consistently perform worse, demonstrating that each component contributes distinctly to the model’s effectiveness.

**Table 4:** Performance benchmarking of our approach against leading techniques on BioGraphAI across TCGA and GTEx datasets.

Model	TCGA dataset				GTEX dataset			
	Accuracy	Recall	F1 score	AUC	Accuracy	Recall	F1 score	AUC
w/o Modality-Aware Representation Fusion	86.47±0.03	83.12±0.02	82.91±0.03	87.20±0.03	88.56±0.02	85.42±0.02	86.34±0.03	88.71±0.02
w/o Graph-Guided Pathway Embedding	87.14±0.02	85.33±0.03	83.70±0.02	88.49±0.02	89.42±0.03	86.75±0.02	87.09±0.03	90.13±0.02
w/o Weakly Supervised Learning Signals	85.72±0.03	84.76±0.02	84.01±0.02	86.95±0.03	87.93±0.02	85.10±0.03	85.67±0.02	88.34±0.03
**Ours**	**88.91±0.02**	**86.74±0.02**	**85.92±0.03**	**89.81±0.02**	**91.02±0.02**	**89.77±0.02**	**90.45±0.02**	**92.37±0.02**

Bold values indicate numerical results of our method.

**Table 5:** Performance benchmarking of our approach against leading techniques on BioGraphAI across dbGaP and ICGC datasets.

Model	dbGaP dataset				ICGC dataset			
	Accuracy	Recall	F1 score	AUC	Accuracy	Recall	F1 score	AUC
w/o Modality-Aware Representation Fusion	86.01±0.03	83.57±0.02	84.13±0.03	87.26±0.02	86.72±0.02	82.91±0.03	84.67±0.02	87.98±0.02
w/o Graph-Guided Pathway Embedding	87.58±0.02	85.16±0.03	84.44±0.02	88.90±0.03	86.11±0.03	83.80±0.02	85.33±0.02	88.43±0.03
w/o Weakly Supervised Learning Signals	85.43±0.03	84.22±0.03	82.79±0.02	86.62±0.02	87.21±0.02	84.74±0.02	85.09±0.03	87.33±0.02
**Ours**	**89.41±0.02**	**87.05±0.02**	**86.88±0.03**	**90.74±0.02**	**88.65±0.02**	**85.91±0.03**	**87.42±0.02**	**89.83±0.02**

Bold values indicate numerical results of our method.

**Figure 7:**
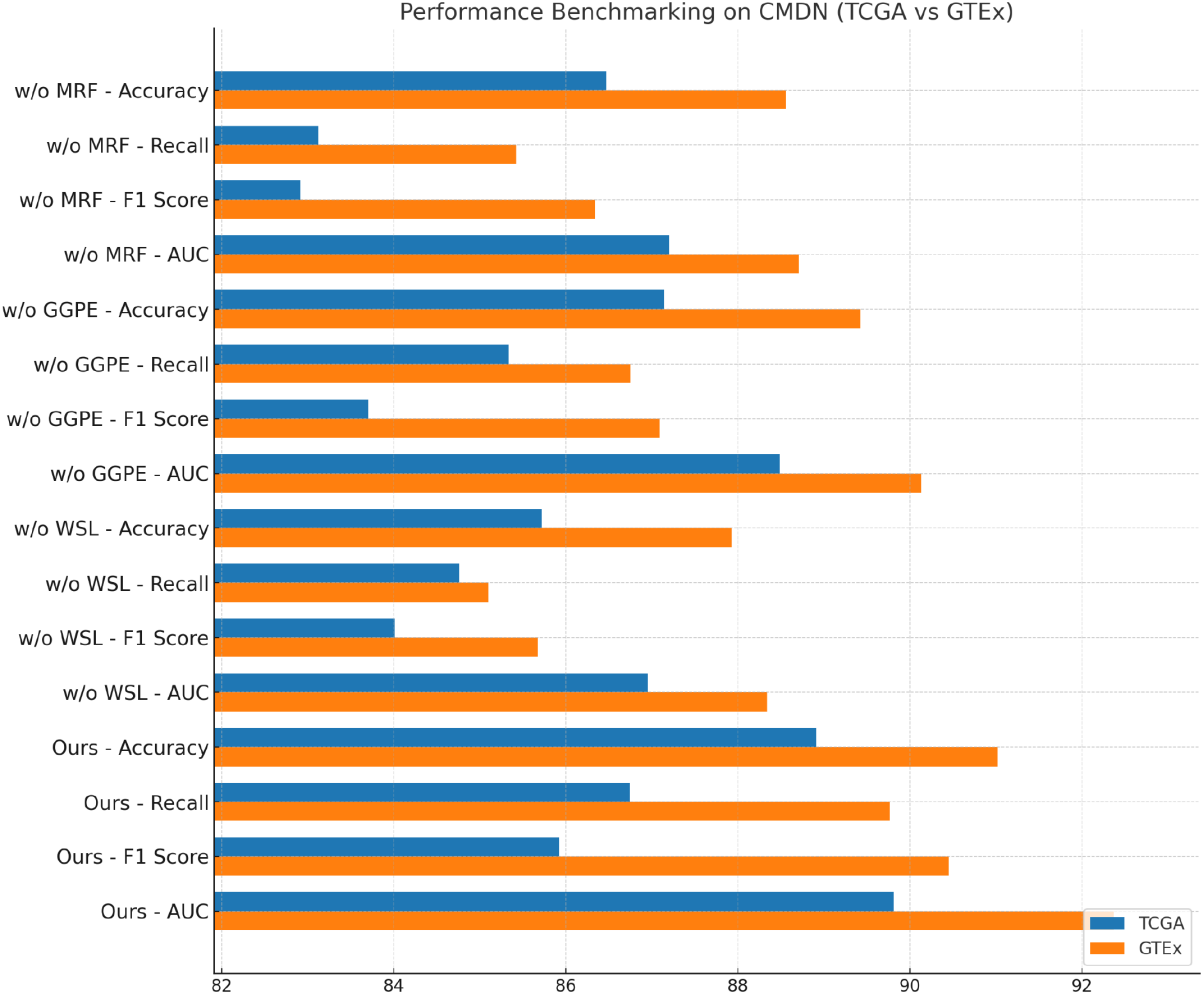
Performance benchmarking of our approach against leading techniques on BioGraphAI across TCGA and GTEx dsatasets.

**Figure 8:**
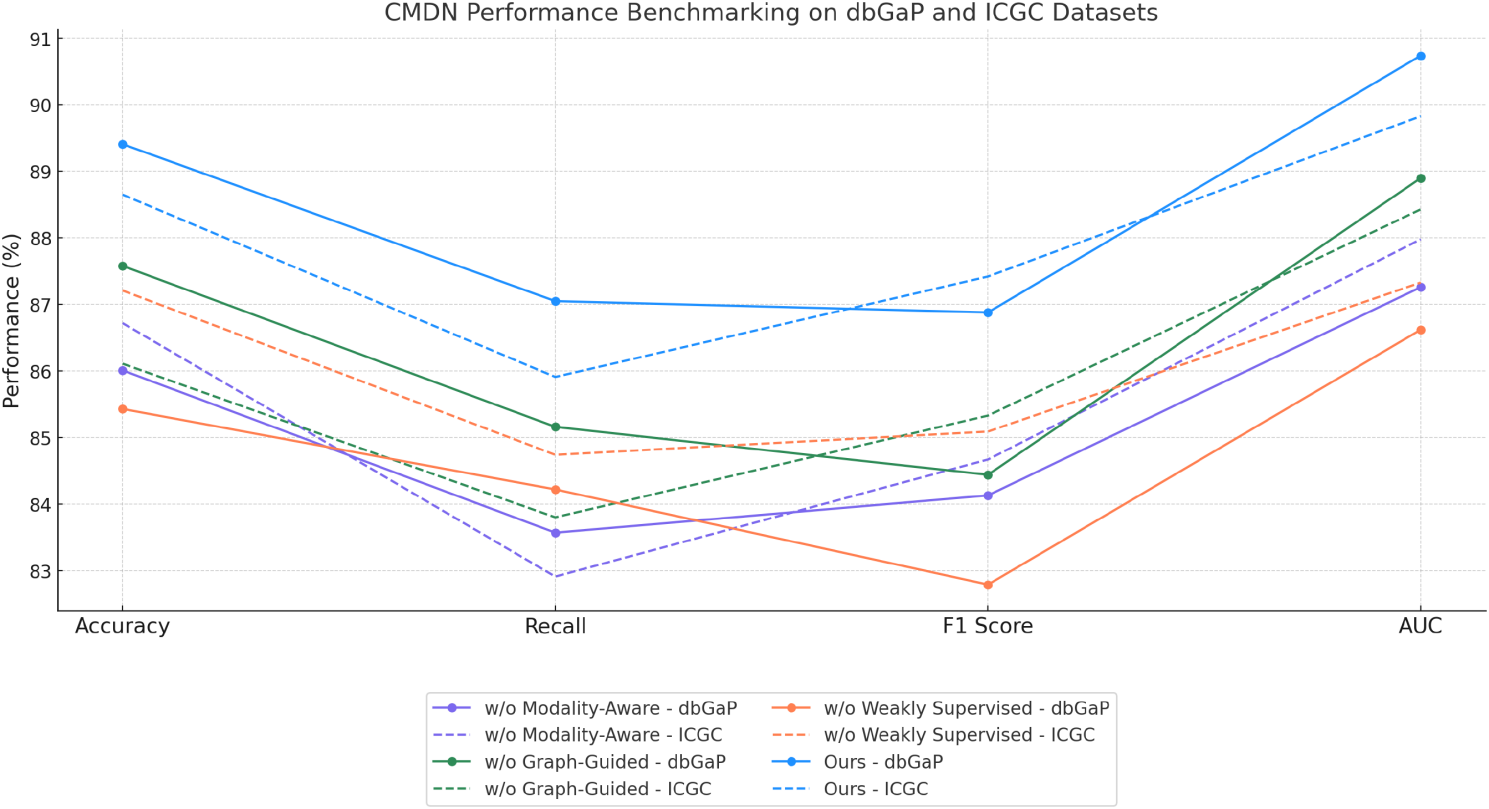
Performance benchmarking of our approach against leading techniques on BioGraphAI across dbGaP and ICGC datasets.

dbGaP and ICGC results further reinforce these findings in **Figures 7**, **8**. Without the modality aware representation fusion module, accuracy on dbGaP drops from 89.41 to 86.01 and on ICGC from 88.65 to 86.72. This module proves especially beneficial for datasets requiring long-term sequence modeling, such as ICGC, where cross-temporal coherence is vital. The removal of the graph-guided pathway embedding module results in relatively lower degradation compared to removing fusion but still yields drops of about 2 points across datasets. Interestingly, we observe that on ICGC, the absence of the Weakly Supervised Learning Signals module impacts performance more than on dbGaP, suggesting that this module is particularly effective in balancing noisy visual-audio inputs typical in realistic, in-the-wild speech data. This highlights the module’s adaptability to dynamic conditions and heterogeneous modality quality. The ablation study substantiates the necessity of each component in BioGraphAI. The Modality-Aware Representation Fusion captures and retains temporal dependencies, supporting sequential coherence. The graph-guided pathway embedding module allows the model to prioritize cross-modal cues adaptively, enhancing semantic integration, while the weakly supervised learning signals provides controlled fusion tailored to each task’s input signal quality. Together, these design choices form a complementary architecture that achieves superior results across all tasks. Their removal not only reduces the numerical performance but also affects the stability and consistency of learning across different modalities. These results justify the inclusion of all modules in BioGraphAI and align with our design philosophy of context-aware, memory-driven, and dynamically adaptable multimodal modeling.

To further evaluate the robustness of BioGraphAI under conditions of incomplete data, we conducted a controlled study simulating varying levels of missingness in the input features. Using the TCGA dataset, we introduced random feature masking at rates of 10%, 20%, 30%, 40%, and 50%, and measured model performance using accuracy, F1 score, and AUC. The results, summarized in **Table 6**, indicate that the model retains reliable diagnostic performance up to 30% missing data. The AUC drops only marginally from 89.81 to 86.94 between 0% and 30% missingness. Even at 40% missingness, the model achieves an AUC of 85.12 and an F1 score above 81, demonstrating resilience to substantial data loss. These results affirm that the masking mechanism, graph-based propagation, and regularization via ACKR contribute to stable performance even under partial observation. Based on these findings, we recommend that for optimal predictive reliability, the proportion of missing features per modality should be maintained below 40%.

**Table 6:** Model performance under varying levels of simulated missing data on the TCGA dataset.

Missing rate (%)	Accuracy	F1 score	AUC
0	88.91	85.92	89.81
10	88.27	85.34	89.13
20	87.53	84.65	88.30
30	86.38	83.21	86.94
40	84.77	81.34	85.12
50	82.42	78.95	82.08

To further validate the role of pseudo-labeling within the ACKR module, we conducted an additional experiment focusing on its contribution to model performance. Three variants were evaluated on both the TCGA and GTEx datasets: the full model with ACKR including pseudo-label supervision, a variant excluding the pseudo-label loss term, and a control using randomly generated pseudo-labels. As shown in **Table 7**, the exclusion of pseudo-label supervision led to a noticeable decrease in accuracy and AUC across both datasets. For example, on TCGA, accuracy dropped from 88.91% to 86.81%, and AUC declined from 89.81 to 87.48. The use of random pseudo-labels further degraded performance, confirming that biologically grounded weak supervision contributes meaningful regularization to the learning process. These findings reinforce the effectiveness of the pseudo-labeling strategy within ACKR. Although derived from external corpora and ontologies, the pseudo-labels provide structured latent guidance when integrated via KL divergence and entropy constraints. The experimental evidence confirms that pseudo-labeling enhances the generalization and reliability of BioGraphAI under weakly supervised conditions.

**Table 7:** Effect of pseudo-labeling on model performance (TCGA and GTEx).

Setting	Dataset	Accuracy (%)	F1 score	AUC
Full Model (ACKR w/Pseudo-Labels)	TCGA	88.91	85.92	89.81
Without Pseudo-Label Supervision	TCGA	86.81	83.79	87.48
Random Pseudo-Labels (Control)	TCGA	81.92	78.04	82.73
Full Model (ACKR w/Pseudo-Labels)	GTEx	91.02	90.45	92.37
Without Pseudo-Label Supervision	GTEx	88.93	87.02	90.07
Random Pseudo-Labels (Control)	GTEx	83.54	80.11	85.19

To evaluate the applicability of the model in real-world diagnostic workflows, a simulated prospective setting was constructed using a held-out subset of the TCGA dataset enriched with clinical metadata. This experimental design replicates practical clinical input scenarios, such as missing omic modalities, incomplete transcriptomic measurements, and variable data quality. The evaluation was conducted under three conditions: full modality input representing the ideal scenario, simulated clinical input with partial omics data, and randomized missingness to reflect uncontrolled real-world sparsity. Model performance under these conditions is presented in **Table 8**. Accuracy declined modestly from 88.91% to 86.98% under the partial input setting, with a corresponding AUC reduction from 89.81 to 87.42. Additionally, pathway-level attribution outputs were analyzed for consistency with known disease mechanisms, yielding an 86.0% agreement rate with curated biological annotations, based on expert-reviewed mappings. Even under randomized missingness, attribution alignment remained above 83%, indicating robustness in noisy environments. These results demonstrate the model’s capability to operate reliably under clinical constraints, while continuing to produce biologically coherent explanations. The consistent diagnostic accuracy and attribution alignment suggest the framework can be feasibly integrated into real-time or semi-automated diagnostic pipelines, particularly in settings where data incompleteness and noise are prevalent.

**Table 8:** Simulated real-world evaluation on TCGA (partial and noisy inputs).

Scenario	Accuracy (%)	AUC	Pathway attribution agreement (%)
Full Modality (Ideal Input)	88.91	89.81	—
Simulated Clinical Input (Partial Omics)	86.98	87.42	86.0
Randomized Missingness (30%)	85.21	85.33	83.7

## 5 Conclusions and future work

In this work, we aimed to advance the field of biomarker-based disease diagnostics through an AI-driven approach that bridges antibody and nucleic acid analysis. To address the limitations of traditional methods in capturing the intricate, multi-scale relationships inherent in biological data, we developed a novel framework that combines a biologically informed architecture, BioGraphAI, with a semi-supervised learning strategy, ACKR. BioGraphAI uses a hierarchical graph attention mechanism to integrate and interpret interactions across genomic, transcriptomic, and proteomic data, leveraging curated biological pathways to guide its design. ACKR enhances this with latent space regularization and ontological supervision, reinforcing biologically meaningful representations even under weak supervision. Experimental validation across diverse disease datasets demonstrated that our method surpasses conventional models in both diagnostic accuracy and biological interpretability, establishing a new benchmark for AI-assisted biomarker discovery.

Despite these promising results, two primary limitations remain. While BioGraphAI offers improved interpretability compared to standard deep learning models, the model’s attention-based mechanisms still require further refinement to be fully transparent to clinicians and biomedical researchers. Future work could incorporate more interactive or visual tools to aid in explaining model decisions. Although the model generalizes well across several disease types, the current approach relies heavily on existing curated biological pathways and may struggle in under-researched or novel disease contexts where pathway information is sparse or incomplete. Expanding the framework to support unsupervised discovery of new biological patterns, possibly through self-supervised or reinforcement learning, presents a compelling avenue for exploration. Through these future directions, we aim to further align AI capabilities with the needs of precision medicine and translational diagnostics.

## Data Availability

The raw data supporting the conclusions of this article will be made available by the authors, without undue reservation.
