# Correction: Artificial intelligence-driven analysis of antibody and nucleic acid biomarkers for enhanced disease diagnostics

**DOI:** 10.3389/fimmu.2025.1727174

**Published:** 2025-10-23

**Authors:** Zihan Liu, Feng Zhu, Mei Zhang

**Affiliations:** ^1^ School of Medicine, Pingdingshan University, Pingdingshan, Henan, China; ^2^ Northeast Agricultural University, Harbin, China

**Keywords:** AI-driven diagnostics, biomarker discovery, antibody and nucleic acid analysis, graph-based modeling, domain knowledge integration

Authors “Zhihan Liu” and “Feng Zhu” were erroneously omitted as equal contributing authors.

The original version of this article has been updated.

